# Cavernous Angioma of the Corpus Callosum Presenting with Acute Psychosis

**DOI:** 10.1155/2014/243286

**Published:** 2014-03-05

**Authors:** Giacomo Pavesi, Francesco Causin, Alberto Feletti

**Affiliations:** ^1^Department of Neurosurgery, Padova Hospital, Via Giustiniani 1, 35100 Padova, Italy; ^2^Department of Neuroradiology, Padova Hospital, Via Giustiniani 1, 35100 Padova, Italy

## Abstract

Psychiatric symptoms may occasionally be related to anatomic alterations of brain structures. Particularly, corpus callosum lesions seem to play a role in the change of patients' behavior. We present a case of a sudden psychotic attack presumably due to a hemorrhagic cavernous angioma of the corpus callosum, which was surgically removed with complete resolution of symptoms. Although a developmental defect like agenesis or lipoma is present in the majority of these cases, a growing lesion of the corpus callosum can rarely be the primary cause. Since it is potentially possible to cure these patients, clinicians should be aware of this association.

## 1. Introduction

Psychiatric manifestations are rarely associated with brain tumors. For this reason, it is often difficult to assess the etiologic role of space-occupying intracranial lesions in the development of psychotic symptoms. Corpus callosum alterations are supposed to increase the risk for behavioral disturbances. However, it is not always possible to exclude the involvement of other surrounding structures. Besides developmental defects like agenesis or lipoma, the association between a well-defined callosal lesion and psychosis is very rare. We report on a patient presenting with acute psychosis associated with a hemorrhagic mid-callosal cavernous angioma.

## 2. Case Report

**Figure 1:**
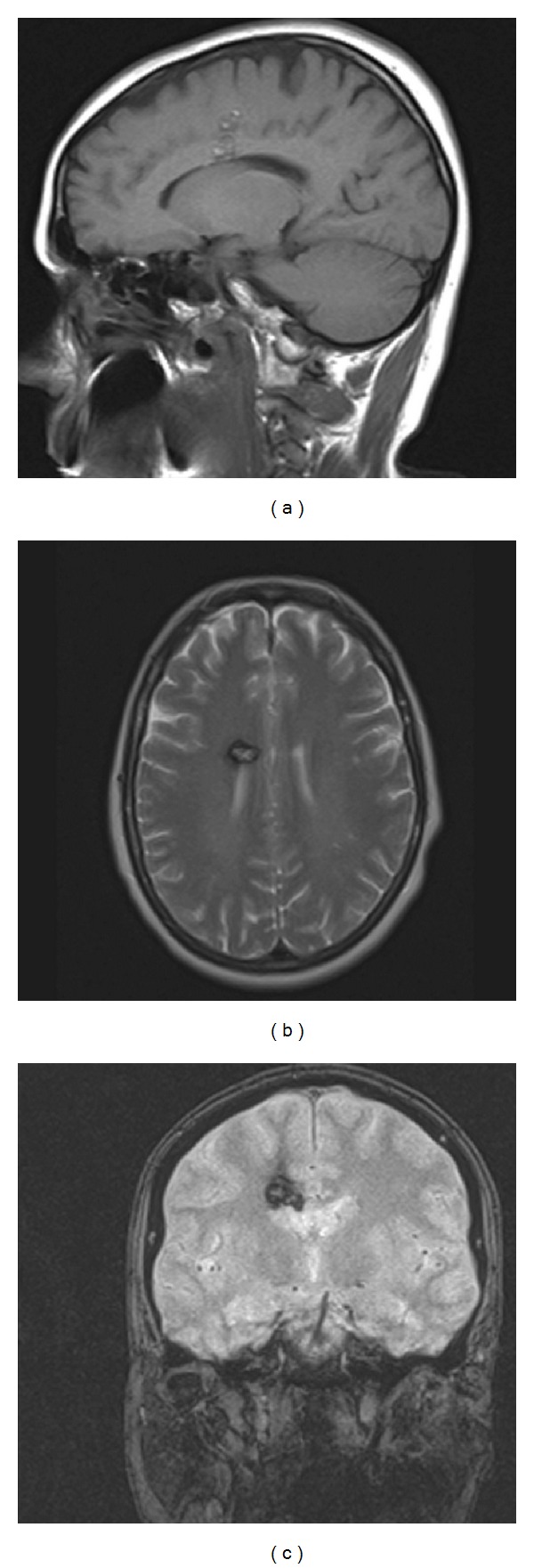
(a) Sagittal T1-weighted MR image showing the cavernous angioma embedded in the middle-third of the corpus callosum and extending into the cingular cortex. Axial T2-weighted (b) and coronal gradient echo (c) MR images evidence signs of a recent intralesional bleeding of the right-sided cavernous angioma.

A 48-year-old Caucasian woman was compulsorily admitted to the psychiatry department of our hospital because of a sudden psychotic event, characterized by persecutory delirium with mystic content. At admission, the patient was suspicious, anxious, and only partially compliant. She had a dysphoric mood, with motor stereotypies. No previous history of mental disease was found. Family history was negative for psychiatric disorders. A pharmacological antipsychotic therapy with intramuscular promazine, lorazepam, and olanzapine was initiated, along with an individual psychotherapy. Consequently, the patient gradually recovered behavioral control and stability in her social and familial relationships. A cerebral MRI showed a cavernous angioma in the middle-third of the corpus callosum, extending upwards to the gyrus cinguli, with signs of a recent intralesional bleeding (Figure 1). The patient was scheduled for elective neurosurgical removal of the lesion. Preoperative neurological exam was normal, besides a mild impairment in recent memory. Microsurgery was performed through an interhemispheric approach. The lesion was removed enbloc from the middle-third of the corpus callosum. Pathologic examination confirmed the diagnosis of cavernous angioma. Postoperative course was uneventful. At 20-month followup the patient was fully recovered and has returned to her previous activities without any residual psychotic manifestation. Antipsychotic medications were discontinued one month after surgery.

## 3. Discussion

The supposed relationship between corpus callosum and behavior is well known. Many authors reported on neuropsychological disorders likely due to lesions involving the corpus callosum [1]. Moreover, the association between major psychiatric disturbance and developmental defects of the corpus callosum has been extensively discussed. Actually, most of the reported cases describe a lipoma of the corpus callosum, which is associated with agenesis in about 50% of cases [2–6]. Some authors hypothesized that defective interhemispheric communication, which is largely mediated by corpus callosum, may underlie schizophrenia [7]. However, the relatively small number of reported cases with schizophrenia and corpus callosum abnormalities, along with the uncertain prevalence of such anomalies in the normal population, does not allow establishing a causal relationship [2, 3]. Also tumors of the corpus callosum can be present with dementia, depression, schizophrenia, and psychosis [8–14]. Usually the tumors are so big that it is not easy to assess whether the primary cause of symptoms is the damage of corpus callosum or the involvement of other adjacent structures. However, in rare cases, psychiatric symptoms are associated with a well-circumscribed lesion of the corpus callosum (Table 1). We report for the first time on a mid-callosal cavernous angioma presenting with a sudden psychotic attack, in the absence of any previous psychiatric history. Apparently, the episode was related to an intralesional bleeding. Brain cavernous angiomas are rare neurovascular lesions. Seizures, focal neurological deficits, and hemorrhage are their most frequent manifestations. Particularly, hemorrhage is the most common cause of an abrupt worsening of symptoms. The patient's gradual recovery over a few weeks is consistent with the typically benign clinical evolution after a low-pressure intracavernoma hemorrhage [15]. These clinical findings along with the MR evidence seem to exclude the presence of overlapping pathologies.

The causal relationship between corpus callosum lesions and psychiatric symptoms is controversial. The recent literature suggests that callosal alterations more likely increase the risk for behavior disturbances, without any direct causative effect. The neurobiological mechanisms underlying the correlation between anatomical location and the psychotic disorder are unknown. Interestingly, patients after transcallosal approach are normally free of psychotic symptoms, although memory and cognitive functions might be impaired [16]. However, the rare cases of well-circumscribed lesions of the corpus callosum with psychosis point out a significant role for interhemispheric disconnection in the development of such symptoms.

## 4. Conclusions

Pathologies affecting the corpus callosum may cause or increase the risk for psychiatric symptoms by interfering with corticocortical interhemispheric connectivity. Both neurosurgeons and psychiatrists should be aware of occasional relationship between isolated psychotic attacks and potentially treatable intracranial lesions. The reported case underscores the importance of conducting a comprehensive neuroradiologic evaluation in patients with psychiatric disturbances.

## Figures and Tables

**Table 1:** Patients with solitary mass lesion of corpus callosum presenting with psychiatric symptoms.

Authors	Age, sex	Clinical features	Psychiatric diagnosis	Organic diagnosis	Treatment	Outcome
Durst and Rosca-Rebaudengo, 1988 [17]	29, m	Acute anxiety and fear of shrinkage/retraction of the penis	Koro syndrome	Tumor of the genu	Electroconvulsive shock therapy (ECT)	Resolution of syndrome

Tanaghow et al., 1989 [18]			Depression	Tumor of anterior part		

Fersten et al., 2001 [19]		Disturbances of emotional-motivation processes, defects in cognitive functions	Affective and paranoid syndromes	PNET of anterior part		

Present case	48, f	Acute delirium with mystic content	Acute psychosis	Cavernous angioma of middle third	Gross total removal	Resolution of symptoms

## References

[1] Devinsky O, Laff R (2003). Callosal lesions and behavior: history and modern concepts. *Epilepsy and Behavior*.

[2] Baumann CR, Regard M, Trier S, Schuknecht B, Siegel AM (2006). Lipoma on the corpus callosum in a patient with schizophrenia-like episode: is there a causal relationship?. *Cognitive and Behavioral Neurology*.

[3] David AS, Wacharasindhu A, Lishman WA (1993). Severe psychiatric disturbance and abnormalities of the corpus callosum: review and case series. *Journal of Neurology Neurosurgery and Psychiatry*.

[4] Gerber SS, Plotkin R (1982). Lipoma of the corpus callosum. Case report. *Journal of Neurosurgery*.

[5] Okumura A, Hayakawa F, Kato T (2009). Callosal lesions and delirious behavior during febrile illness. *Brain and Development*.

[6] Pinkofsky HB, Struve FA, Meyer MA, Patrick G, Reeves RR (1997). Decreased multi-band posterior interhemispheric coherence with a lipoma on the corpus callosum: a case report of a possible association. *Clinical Electroencephalography*.

[7] Chaim TM, Schaufelberger MS, Ferreira LK (2010). Volume reduction of the corpus callosum and its relationship with deficits in interhemispheric transfer of information in recent-onset psychosis. *Psychiatry Research*.

[8] Filley CM, Kleinschmidt-DeMasters BK (1995). Neurobehavioral presentations of brain neoplasms. *Western Journal of Medicine*.

[9] Harrison MJG (1984). Dementia due to tumours of the corpus callosum. *Postgraduate Medical Journal*.

[10] Lisanby SH, Kohler C, Swanson CL, Gur RE (1998). Psychosis secondary to brain tumor. *Seminars in Clinical Neuropsychiatry*.

[11] Monaco EA, Armah HB, Nikiforova MN, Hamilton RL, Engh JA (2011). Grade II oligodendroglioma localized to the corpus callosum. *Brain Tumor Pathology*.

[12] Osawa A, Maeshima S, Kubo K, Itakura T (2006). Neuropsychological deficits associated with a tumour in the posterior corpus callosum: a report of two cases. *Brain Injury*.

[13] Ouma JR (2004). Psychotic manifestations in brain tumour patients: 2 case reports from South Africa. *African Health Sciences*.

[14] Wessling H, Simosono CL, Escosa-Bagé M, de Las Heras-Echeverría P (2006). Anton’s syndrome due to a giant anterior fossa meningioma. The problem of routine use of advanced diagnostic imaging in psychiatric care. *Acta Neurochirurgica*.

[15] Kivelev J, Niemelä M, Kivisaari R, Dashti R, Laakso A, Hernesniemi J (2009). Long-term outcome of patients with multiple cerebral cavernous malformations. *Neurosurgery*.

[16] Peltier J, Roussel M, Gerard Y (2012). Functional consequences of a section of the anterior part of the body of the corpus callosum: evidence from an interhemispheric transcallosal approach. *Journal of Neurology*.

[17] Durst R, Rosca-Rebaudengo P (1988). Koro secondary to a tumour of the corpus callosum. *British Journal of Psychiatry*.

[18] Tanaghow A, Lewis J, Jones GH (1989). Anterior tumour of the corpus callosum with atypical depression. *British Journal of Psychiatry*.

[19] Fersten E, Łuczywek E, Głowacki M, Czernicki Z (2001). Paranoid syndrome in a patient with tumor in anterior part of corpus callosum. Case report. *Neurologia i Neurochirurgia Polska*.

